# Periodontal Treatment Protocol for Decompensated Diabetes Patients

**DOI:** 10.3389/froh.2021.666713

**Published:** 2021-04-16

**Authors:** Matías Dallaserra, Alicia Morales, Nayib Hussein, Marcela Rivera, Franco Cavalla, Mauricio Baeza, Franz J. Strauss, Yazmin Yoma, Claudio Suazo, Gisela Jara, Johanna Contreras, Julio Villanueva, Francisca Valenzuela-Villarroel, Jorge Gamonal

**Affiliations:** ^1^Department of Oral and Maxillofacial Surgery, Faculty of Dentistry, University of Chile, Santiago, Chile; ^2^Cochrane Associate Center, Faculty of Dentistry, University of Chile, Santiago, Chile; ^3^Center for Epidemiology and Surveillance of Oral Diseases (CESOD), Faculty of Dentistry, University of Chile, Santiago, Chile; ^4^Department of Conservative Dentistry, Faculty of Dentistry, University of Chile, Santiago, Chile; ^5^Centro de Salud Familiar Dr. Francisco Boris Soler, Melipilla, Chile; ^6^Departamento de Atención de las Personas, División de Atención Primaria, Subsecretaría de Redes Asistenciales del Ministerio de Salud, Santiago, Chile; ^7^Clinic of Reconstructive Dentistry, Center of Dental Medicine, University of Zurich, Zurich, Switzerland; ^8^Department of Oral Biology, Medical University of Vienna, Vienna, Austria; ^9^Referencia Técnica Odontológica, Departamento de Gestión Clínica, Servicio de Salud Metropolitano Occidente, Santiago, Chile; ^10^Programa Odontológico, Cesfam Steeger, Corporación Municipal Desarrollo Social de Cerro Navia, Santiago, Chile; ^11^Servicio de Cirugía Maxilofacial, Hospital Clínico San Borja Arriarán, Santiago, Chile; ^12^Faculty of Medicine, University of Chile, Santiago, Chile

**Keywords:** periodontitis, diabetes mellitus, periodontal therapy, protocol, glycated (glycosylated) hemoglobin

## Abstract

**Background:** Decompensated diabetes is associated with a higher prevalence and severity of periodontitis and poorer response to periodontal therapy. It is conceivable that periodontal therapy may cause systemic and local complications in this type of patients. The aim of the present study was to identify and describe the best available evidence for the treatment of periodontitis in decompensated diabetics.

**Material and methods:** An expert committee including participants from different areas gathered to discuss and develop a treatment guideline under the guidance of the Cochrane Associate Center, Faculty of Dentistry, University of Chile. In total, four research questions were prepared. The questions prepared related to decompensated diabetic patients (glycated hemoglobin >8) were, (1) Does the exposure to periodontal treatment increase the risk of infectious or systemic complications? (2) Does the antibiotic treatment or prophylaxis, compared to not giving it, reduce infectious complications? (3) Does the exposure to periodontal treatment, compared to no treatment, reduce the glycated hemoglobin levels (HbA1c)? Last question was related to diabetic patients, (4) Does the exposure to a higher level of HbA1c, compared to stable levels, increase the risk of infectious complications? Based on these questions, a search strategy was developed using MEDLINE and EPISTEMONIKOS. Only systematic reviews were considered.

**Results:** For question 1, the search yielded 12 records in EPISTEMONIKOS and 23 in MEDLINE. None of these studies addressed the question. For question 2, the search yielded 58 records in EPISTEMONIKOS and 11 in MEDLINE. None of these studies addressed the question. For question 3, the search yielded 16 records in EPISTEMONIKOS and 11 in MEDLINE. Thirteen addressed the question. For question 4, the search yielded 7 records in EPISTEMONIKOS and 9 in MEDLINE. One addressed the question.

**Conclusions:** In decompensated diabetic patients, there is lack of scientific information about risk of infectious or systemic complications as a result of periodontal treatment and about the impact of antibiotic treatment or prophylaxis on reduction if infectious complications. A defined HbA1c threshold for dental and periodontal treatment in diabetic patients has yet to be determined. Finally, periodontal treatment does have an impact on HbA1c levels.

## Introduction

Chronic non-communicable diseases (NCDs) are a worldwide public health issue, and the leading cause of death with an estimated of 41 million deaths per year [1]. In Chile, it is estimated that 22.8% of the population suffers from two or more NCDs. Diabetes is one of the most frequent NCDs in the population, with a prevalence of 382 million worldwide in 2013 that would increase to 592 million in 2035 [2]. According to the latest data obtained from the National Health Survey [3], Chile has a prevalence of diabetes of about 12%.

Among oral diseases, non-treated caries in permanent teeth is the most prevalent condition worldwide, affecting 2.5 billion people; non-treated caries in primary teeth affect 573 million children, and severe periodontal disease affects 538 million people, including 276 million people with total tooth loss [4]. In Chile, periodontitis affects more than two thirds of the adult population [5, 6]. Periodontitis has been mentioned as the sixth complication of diabetes, with higher degrees of severity in patients with poor glycemic control of their diabetes [7, 8].

Several studies show the bidirectional relationship existing between diabetes and periodontitis. Their connection lies in the common underlying inflammatory processes. When decompensated, diabetes acts as a predisposing factor for periodontitis, affecting the response to periodontal treatment and increasing the risk of tooth loss [9–11]. On the other hand, it has been demonstrated that periodontitis has a deleterious effect on metabolic control in diabetic patients, aggravating cardiovascular complications [9, 10].

In patients with diabetes and periodontitis, improvement in the metabolic indicators of diabetes associated with periodontal treatment still shows contradictory results. Whereas, some studies demonstrate a beneficial effect by lowering the glycemic and glycated hemoglobin levels [12–15], other studies have concluded that there is insufficient evidence for a beneficial effect [16, 17].

In the Chilean Primary Health Care (PHC), a model of comprehensive care has been implemented, in which one of the central aims is the treatment of NCDs [18]. As a result, different health programs exist, in order to cover all the diseases affecting the Chilean population; among them is the Cardiovascular Health Program (CVHP) [19]. It consists of the comprehensive and multidisciplinary care of patients suffering from cardiovascular risk factors who enter in a follow-up and control system, with the aim to decrease the risk of dying. Individuals diagnosed with one or more of the following conditions are admitted in the program: history of atherosclerotic disease, dyslipidemia, arterial hypertension, type 2 diabetes mellitus, and smoking in people older than 55. Reference criteria of determined complications are included in the care of diabetic patients, in which the timely investigation of retinopathy, nephropathy, macroangiopathy and diabetic foot is encouraged [19]. PHC however, lacks a protocol for the care of patients with periodontitis. This results in an increase of referrals to the hospital rising the workload on the specialist and creating a waiting list for periodontal treatment. It should be emphasized that the treatment of periodontitis is included in the list of benefits of the different dental programs existing in the PHC [20–22].

Although the bidirectional relationship of the two diseases has been demonstrated, periodontal treatment is not included within the CVHP for diabetic patients. Moreover, the clinical guidelines for periodontal disease do not define clear directions regarding the management of a decompensated diabetic patient, often resulting in delayed dental care which would ultimately improve the health conditions of the patients [23].

Primary health care must consider the effect of periodontal treatment on the metabolic control of diabetic patients. Therefore, dentistry has the challenge of integrating into a patient-centered care, where clinical care is approached by multiple professionals belonging to different healthcare areas, with fluent communication and focusing on the resolution of the diseases as a multidisciplinary team [24]. Therefore, the aim of the present study was to identify and describe the best available evidence for the treatment of periodontitis in patients with decompensated diabetes and thereby develop a treatment protocol.

## Materials and Methods

### Study Design

The design of this study is a critical review of the scientific literature related to the topic, with the objective to identify and describe the best available evidence on a number of questions already established. EPISTEMONIKOS was used for the search of information, as well as the MEDLINE database. EPISTEMONIKOS combines the best Evidence-Based Health Care, information technologies and a network of experts to provide a unique tool for people making decisions concerning clinical or health-policy questions.

### Expert Committee and Preparation of Questions to Investigate

Before the beginning of the study, a group of experts was created, with representatives from the area of periodontal research (AM, MB, FC, FS, JG), public health (CS, YY, GJ), primary health care (CS, YY), family medicine (MR, FVV) and the Cochrane Associate Center of the Faculty of Dentistry, Universidad de Chile (MD, JV). The objective of this group of experts was to gather the main concerns and uncertainties in the dental and medical field, in relation to the periodontal management of patients with diabetes mellitus, with emphasis on the Primary Health Care Programs existing in Chile, in order to prepare the questions that, once resolved, will allow us to propose protocols of care.

In total, four research questions were prepared. The questions prepared related to decompensated diabetic patients (glycated hemoglobin >8) were, [1] does the exposure to periodontal treatment increase the risk of infectious or systemic complications? [2] Does the antibiotic treatment or prophylaxis, compared to not giving it, reduce infectious complications? [3] Does the exposure to periodontal treatment, compared to no treatment, reduce the glycated hemoglobin levels (HbA1c)? Last question was related to diabetic patients, [4] Does the exposure to a higher level of HbA1c, compared to stable levels, increase the risk of infectious complications?

### Search of Information and Inclusion Criteria

From these questions, search strategies were built for the EPISTEMONIKOS database, as well as for the MEDLINE database, in order to confirm the results. MeSH terms used in the search strategy are described in Table 1. Inclusion criteria for this study are the systematic reviews answering directly the questions involved, since the objective is to identify and describe the best available evidence for each question. All the studies selected were evaluated by independent experts (NH, MD, and JV).

**Table 1:** Search strategies.

**Question**	**EPISTEMONIKOS**	**MEDLINE**
Question 1	[(“diabetes” AND “mellitus”) OR “diabetes mellitus” OR “diabetes” OR (“diabetes” AND “insipidus”) OR “diabetes insipidus”)] AND [periodontal AND (“therapy” OR “treatment” OR “therapeutics”)] OR (“dental” AND “scaling”) OR “dental scaling” OR (“root” AND “scaling”) OR “root scaling” OR (“root” AND “planning”) OR “root planning”)	(“diabetes mellitus”[MeSH Terms] OR (“diabetes”[tiab] AND “mellitus”[tiab]) OR “diabetes mellitus”[tiab] OR “diabetes”[tiab] OR “diabetes insipidus”[MeSH Terms] OR (“diabetes”[tiab] AND “insipidus”[tiab]) OR “diabetes insipidus”[tiab]) AND (periodontal[tiab] AND (“therapy”[Subheading] OR “therapy”[tiab] OR “treatment”[tiab] OR “therapeutics”[MeSH Terms] OR “therapeutics”[tiab])) OR (“dental scaling”[MeSH Terms] OR (“dental”[tiab] AND “scaling”[tiab]) OR “dental scaling”[tiab] OR (“root”[tiab] AND “scaling”[tiab]) OR “root scaling”[tiab]) OR (“root planning”[MeSH Terms] OR (“root”[tiab] AND “planning”[tiab]) OR “root planning”[tiab]) AND (“complications”[Subheading] OR “complications”[tiab])
Question 2	((“diabetes” AND “mellitus”) OR “diabetes mellitus” OR “diabetes” OR (“diabetes” AND “insipidus”) OR “diabetes insipidus” or diabetic*) AND (periodontal AND (“therapy” OR “treatment” OR “therapeutics”) OR (“dental” AND “scaling”) OR “dental scaling” OR (“root” AND “scaling”) OR “root scaling” OR (“root” AND “planning”) OR “root planning”) AND (antibiotic* OR antimicrobial*)	((“diabetes mellitus”[MeSH Terms] OR (“diabetes”[tiab] AND “mellitus”[tiab]) OR “diabetes mellitus”[tiab] OR “diabetes”[tiab] OR “diabetes insipidus”[MeSH Terms] OR (“diabetes”[tiab] AND “insipidus”[tiab]) OR “diabetes insipidus”[tiab]) AND (“dental scaling”[MeSH Terms] OR (“dental”[tiab] AND “scaling”[tiab]) OR “dental scaling”[tiab] OR (“root”[tiab] AND “scaling”[tiab]) OR “root scaling”[tiab] OR (“root planning”[MeSH Terms] OR (“root”[tiab] AND “planning”[tiab]) OR “root planning”[tiab])) AND (“antibiotic prophylaxis”[MeSH Terms] OR “anti-bacterial agents”[MeSH Terms] OR (“antibiotic”[tiab] AND “prophylaxis”[tiab]) OR “antibiotic prophylaxis”[tiab] OR antibiotic*[tiab]))
Question 3	(“diabetes mellitus” OR (“diabetes” AND “mellitus”) OR “diabetes insipidus” OR (“diabetes” AND “insipidus”) OR “diabetes insipidus”) AND “periodontitis” AND [(“dental scaling” OR (“dental” AND “scaling”) OR (“root” AND “scaling”) OR “root scaling”) OR (“root planning” OR (“root” AND “planning”) OR “root planning”)] AND ((“glycated hemoglobin a” OR “hba1c”) OR (“metabolic control” OR (“metabolic” and “control”) OR “control”))	(“diabetes mellitus”[MeSH Terms] OR (“diabetes”[tiab] AND “mellitus”[tiab]) OR “diabetes mellitus”[tiab] OR “diabetes”[tiab] OR “diabetes insipidus”[MeSH Terms] OR (“diabetes”[tiab] AND “insipidus”[tiab]) OR “diabetes insipidus”[tiab]) AND (“periodontitis”[MeSH Terms] OR “periodontitis”[tiab]) AND ((“dental scaling”[MeSH Terms] OR (“dental”[tiab] AND “scaling”[tiab]) OR “dental scaling”[tiab] OR (“root”[tiab] AND “scaling”[tiab]) OR “root scaling”[tiab]) OR (“root planning”[MeSH Terms] OR (“root”[tiab] AND “planning”[tiab]) OR “root planning”[tiab])) AND (“glycated hemoglobin a”[MeSH Terms] OR “glycated hemoglobin a”[TIAB] OR “hba1c”[TIAB] OR ((metabolic[tiab] AND “control”[tiab]) OR “control”[tiab]))
Question 4	(“diabetes mellitus” OR (“diabetes” AND “mellitus”) OR “diabetes” OR “diabetes insipidus” OR (“diabetes” AND “insipidus”)) AND (“glycated hemoglobin a” OR “hba1c”) AND (infect* AND complication*)	(“diabetes mellitus”[MeSH Terms] OR (“diabetes”[tiab] AND “mellitus”[tiab]) OR “diabetes mellitus”[tiab] OR “diabetes”[tiab] OR “diabetes insipidus”[MeSH Terms] OR (“diabetes”[tiab] AND “insipidus”[tiab]) OR “diabetes insipidus”[tiab]) AND (“glycated hemoglobin a”[MeSH Terms] OR “glycated hemoglobin a”[TIAB] OR “hba1c”[TIAB]) AND (infect*[tiab] AND “complications”[tiab])

For the questions that had systematic reviews matching the inclusion criteria, evidence matrixes were built in EPISTEMONIKOS to identify the reviews answering the question and the primary studies included. Afterwards, a descriptive report of the available evidence was made for these cases, in relation to the reported effects, risk of bias and methodological aspects of the design of the included studies and their respective primary studies. Regarding the questions without results matching the inclusion criteria, future methodological recommendations will be suggested, according to the type of question.

## Results

The results of the search strategy for the 4 questions, according to EPISTEMONIKOS and MEDLINE, are found in Table 2.

**Table 2:** Results of the search strategies.

**Question**	**Results EPISTEMONIKOS (total/relevant)**	**Results MEDLINE**	**Total relevant/included in the matrix**
1	12/0	23/0	0
2	58/0	11/0	0
3	16/10	11/6	8/13
4	7/1	9/1	1

When searching studies for the following question: “In decompensated diabetic patients (HbA1c >8%), does the exposure to periodontal treatment increase the risk of infectious or systemic complications?,” a total of 12 systematic reviews was obtained in EPISTEMONIKOS, none of which was relevant for the question asked. While the search strategy for the same question in MEDLINE, gave a total of 23 systematic reviews, none of which was relevant for the question asked, confirming the results of EPISTEMONIKOS.

When searching studies for the following question: “In decompensated diabetic patients (HbA1c >8%), does the antibiotic treatment or prophylaxis, compared to not giving it, reduce infectious complications?,” the search strategy in EPISTEMONIKOS for this question obtained 58 systematic reviews, none of which was relevant for the structures question asked. On the other hand, the search strategy for the same question in MEDLINE obtained 11 systematic reviews, none of which answered the question asked, also confirming the results of EPISTEMONIKOS.

**Figure 1:**
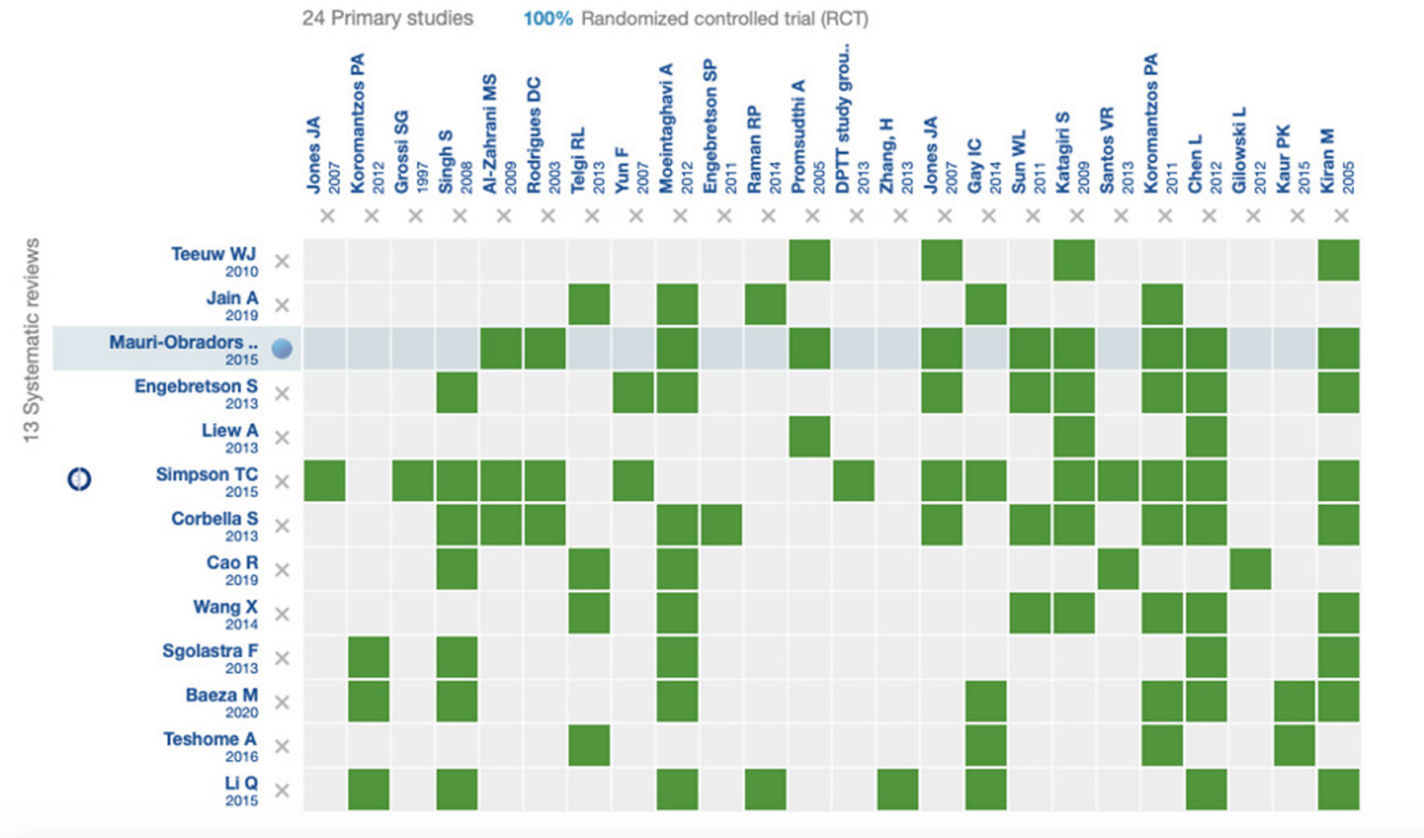
Evidence matrix including the systematic reviews answering question 3, and their respective randomized clinical trials.

In the next question: “In decompensated diabetic patients (HbA1c >8%), does the exposure to periodontal treatment, compared to no treatment, reduce the HbA1c?,” the search strategy applied in EPISTEMONIKOS and MEDLINE allowed for the construction of an evidence matrix with 13 systematic reviews [12, 17, 25–35] (Figure 1). The systematic reviews contained in the matrix built for this question included 37 primary studies, of which 24 are randomized clinical trials. The effect of periodontal therapy on the levels of HbA1c reported in the different meta-analyses varied with mean differences from 0.27 to 0.65, with statistically significant and clinically relevant results, in favor of the intervention [12, 17, 25–28, 30, 32, 35]. Tables 3, 4 show the description of the systematic reviews included in the question.

**Table 3:** Reviews included in questions 3 and 4.

**Question**	**Relevant in EPISTEMONIKOS**	**Relevant in MEDLINE**
3	[25] [26] [12] [27] [28] [29] [17] [33] [34] [35]	[30] [31] [17] [32] [33] [34]
4	[36]	[36]

**Table 4:** Description of reviews included in question 3.

**Review**	**Inclusion criteria**	**Population**	**Intervention/control**	**Outcomes**	**Studies included**	**Risk of bias, studies included**	**Results**	**Observations**
Teeuw et al. [25]	(1) Study design: Original investigation (CCT or RCT) (2) Population: Diabetic patients with periodontitis (3) Intervention: Periodontal treatment (4) Comparison: No treatment (5) Outcome: Metabolic control (HbA1c or FPG) (6) Follow- up: ≥3 months	Diabetic patients with periodontitis.	-Intervention: Periodontal treatment. - Control: without periodontal treatment	Absolute change in HbA1c (%)	5 (3 RCT y 2 CCT)	3 RCT with low risk of bias. 2 CCT with moderate risk of bias.	HbA1c (%): WMD −0.40 [−0.77; −0.04] *I*^2^: 59,5%	Publication bias: does not comply with search in minimum number of bases, articles only in English. They do not present the risk of bias assessment of the included studies; they only present an overall assessment of each study.
Corbella et al. [26]	(1) Study design: RCT (2) Population: diabetic patients with periodontal disease (3) Outcome: HbA1c and/or modification of FPG (4) Follow- up: ≥3 months (5) Clear presentation of population demographic characteristics	Patients with diabetes and periodontal disease.	- Intervention: Nonsurgical periodontal treatment. - Control: without periodontal treatment.	Absolute change in the percentage of HbA1c.	15 RCT	Risk of bias assessment with Cochrane tool was positive for most of the studies, where a smaller percentage has a high risk of bias, especially at the level of the allocation concealment.	HbA1c (%): Analysis of subgroup of studies with low risk of bias: WMD: −0.32[−0.44; −0.19] *I*^2^: 42% Analysis of subgroup of studies with moderate risk of bias: WMD: −0.80 [−1.17; −0.44] *I*^2^: 0% Complete analysis: WMD: −0.38 [−0.53; −0.23] *I*^2^: 50%	Review with a good methodological quality. Does not present a level of certainty of evidence.
Engebretson and Kocher [12]	(1) Study design: RCT (2) Population: diabetic patients (DM1 or DM2), aged >18 years. Diagnosed with periodontitis. (3) Intervention: periodontal therapy (4) Comparison: no treatment or delayed treatment (5) Outcome: HbA1c, or FPG, or OGTT, as the primary outcome. (6) Follow- up: ≥3 months (7) Published in English.	Patients with DM1 or DM2 and periodontitis	- Intervention: Non-surgical periodontal treatment or surgical treatment with or without the use of adjuvant antibiotics or any other use of drugs (anti-inflammatory). - Control: No treatment or delayed treatment	Absolute change from baseline HbA1c (%)	9 RCT	Mentions risk of bias assessment but does not specify it in the results.	HbA1c (%): WMD: −0.36 [−0.54; −0.19] *I*^2^: 9%	Publication bias: does not comply with search in minimum number of bases, articles only in English.
Liew et al. [27]	(1) Study design: RCT. (2) Population: diabetic patients (DM2), aged ≥16 years-old. diagnosed with periodontitis. (3) Intervention: non-surgical periodontal treatment. (4) Comparison: without periodontal treatment or delayed treatment. (5) Outcome: mean change in HbA1c level; or HbA1c levels before and after treatment. (6) Follow-up: ≥3 months (7) Published in English.	Individuals with diabetes mellitus and periodontal disease, aged at least 16.	-Intervention: Non-surgical periodontal treatment (mechanical instrumentation, ultrasonic debridement, supragingival irrigation, subgingival irrigation; with or without complementary local administration of drugs and systemic antibiotics). - Control: No treatment or delayed treatment	Absolute change in the percentage of HbA1c before treatment.	6 RCT	Studies included presented a threat to the blind internal validity, reporting allocation concealment and loss data management.	HbA1c (%): WMD: −0.41 [−0.73; −0.09] *I*^2^: 57.8%	Publication bias: articles only in English. Asymmetric distribution in Funnel plot.
Sgolastra et al. [28]	(1) Study design: RCT. (2) Population: adult patients (>18 years old) diagnosed with DM2 and CP; (3) Intervention: Non-surgical periodontal treatment. (4) Comparison: No treatment, coronal SRP or mechanical dental cleaning; (5) Outcome: Pre- and post- change in HbA1c. (6) Follow-up: ≥3 months	Patients with CP and DM2	-Intervention: scaling and root planning. - Control: no treatment, supragingival scaling or mechanical dental cleaning	The primary outcomes were changes in HbA1c (%) and FPG (mg/dL).	5 RCT	3 RCT: high risk of bias2 RCT: low risk of bias. However, the tool used is not the one suggested by the PRISMA statement.	HbA1c (%): WMD: −0.65 [−0.88; −0.43] *I*^2^: 0%	High risk of bias in primary studies included.
Wang et al. [29]	(1) Study design: RCT (2) Population: Subjects diagnosed with DM2 and periodontitis. (3) Intervention: Periodontal treatment combined with systemic antibiotics. (4) Comparison: SRP alone or SRP plus placebo. (5) Outcome: Mean change in HbA1c level.	Participants DM2 and periodontitis	-Intervention: SRP with administration of oral doxycycline - Control: SRP alone or SRP plus placebo	Absolute change in the percentage of HbA1c	4 RCT	Hig risk of bias in most of the studies, especially the blind.	HbA1c (%): WMD: −0.23 [−0.61; 0.14] *I*^2^: 0%	High risk of bias of the primary studies included.
Li et al. [30]	(1) Study design: RCT (2) Population: patients diagnosed with DM2 and periodontitis. (3) Intervention: Non-surgical treatment. (4) Comparison: no periodontal treatment or delayed treatment. (5) Outcome: Mean change in HbA1c level. (6) Follow- up: ≥3 months (7) Published in English.	Patients with DM2 and periodontitis.	-Intervention: non-surgical periodontal treatment without complementary local administration of drugs and systemic antibiotics. - Control: no periodontal treatment or delayed treatment.	Mean change in HbA1c levels	9 RCT	Only two articles reported intention-to-treat analysis and no allocation concealment is observed in 3 articles.	HbA1c: WMD: −0.27 [-0.46; −0.07]. *I*^2^: 41.7%	Publication bias: only articles in English included.
Mauri-Obradors et al. [31]	(1) Study design: RCT (2) Population: Subjects diagnosed with DM1 or DM2 and periodontitis (*n* > 20) (3) Intervention: Non-surgical periodontal treatment. (4) Comparison: No treatment (5) Outcome: Mean change in HbA1c level. (6) Follow- up: ≥3 months (7) Published in English (2001–2012)	Patients with Type 2 diabetes and periodontal disease.	Intervention: Scaling and root planning. Control: without periodontal treatment.	Absolute change in the percentage of HbA1c before treatment.	21 (13 RCT and 8 CCT)	Does not use tools for risk of bias assessment, recommended py PRISMA. Uses the Jadad scale.	Qualitative systematic review, no meta-analysis was carried out. Does not justify why.	Publication bias: does not comply with search in minimum number of bases, articles only in English.
Simpson et al. [17]	(1) Study design: RCT (2) Population: People with DM1 or DM2 and periodontitis (3) Intervention: Periodontal treatment (4) Comparison: No treatment or alternative periodontal therapy (5) Outcome: Mean change in HbA1c level. (6) Follow- up: ≥3 months	Individuals with DM1 or DM2 and periodontitis	-Intervention: Periodontal treatments (mechanical debridement, surgical treatment and antimicrobial therapy). - Control: No active intervention/usual care or alternative periodontal therapy	Absolute change in the percentage of HbA1c before treatment and 90 days after treatment.	35 RCT included in the qualitative analysis.	The internal validity of the studies included presented a threat in most of the bias criteria. The results have a poor level of certainty of evidence because of the high risk of bias of the primary studies (especially the blind) and a moderate heterogeneity (53%).	HbA1c (%): WMD: −0.29 [−0.48; −0.10] *I*^2^: 53%	Review with a good methodological quality. The results are only threatened by the internal validity of the primary studies included.
Teshome and Yitayeh [32]	(1) Study design: RCT (2) Population: Patients with DM2 and periodontitis (3) Intervention: Non-surgical periodontal treatment (4) Comparison: No periodontal treatment (5) Outcome: Mean change in HbA1c level or FPG (6) Published in English.	Patients with DM2 and periodontitis	-Intervention: Non-surgical periodontal treatment with/without adjuvant antibiotics - Control: No periodontal treatment.	Changes in HbA1c (expressed in %) and fasting plasma glucose (FPG) (expressed in mg/dL).	7 RCT	All articles reported incomplete outcome data status, intention-to-treat analysis, and report selection. Generation of random sequences and allocation concealment were not clearly established in two studies.	HbA1c (%): WMD: −0.48 [−0.78; −0.18] *I*^2^: 99%	Publication bias: only articles in English included. Consistency of the study is low, because of its high level of heterogeneity.
Cao et al. [33]	(1) Study design: RCT (2) Population: Adult patients (≥30 years old) and diagnosed with periodontitis and DM2 (3) Intervention: Periodontal treatment (4) Comparison: No treatment, SRP or adjuvant therapies. (5) Outcome: Mean change in HbA1c or FPG (6) Follow- up: ≥3 months (7) Published in English.	Adult patients (≥30 years old) diagnosed with periodontitis and DM2	-Intervention: SRP, SRP plus adjuvant treatment, different adjuvant therapies - Control: No treatment, SRP, SRP plus adjuvant therapies.	Absolute change in HbA1c (%)	14 RCT	Most of the studies had methodological problems. The most problematic domain was allocation concealment (uncertain or high risk in 35.7% of the studies)	HbA1c (%): Meta-analysis results (SRP vs. NT): WMD: −0.45 [−0.89; 0.00] Network meta-analysis results (SRP vs. NT): WMD: −0.40 [−0.80; −0.08) (0.088, 0.80)	Publication bias: only articles in English included. Does not comply with search in minimum number of bases. High risk of bias of the primary studies included.
Jain et al. [34]	(1) Study design: RCT (2) Population: Patients diagnosed with CP and DM2 (3) Intervention: SRP (4) Comparison: No treatment (5) Outcome: Mean change in HbA1c (6) Follow-up: (7) Published in English (2006–2016)	Patients with DM2 and CP	-Intervention: SRP without any supportive use of local drug delivery and systemic antibiotics - Control: No periodontal treatment.	Mean change in HbA1c (%)	6 RCT	Most of the studies showed low risk of bias for almost all parameters evaluated, while 30% of the studies showed a high risk of performance and detection bias. In addition, the risk of bias was not clear for selection bias (allocation concealment).	HbA1c (%): WMD: −0.26 [−0.63; 0.11] *I*^2^: 84%	Publication bias: does not comply with search in minimum number of bases, articles only in English.
Baeza et al. [35]	(1) Study design: RCT (2) Population: People with DM2 and periodontitis (3) Intervention: Periodontal treatment (4) Comparison: No periodontal treatment (5) Outcome: Mean change in HbA1c, CRP. (6) Follow-up: ≥3 months	Patients DM2 and periodontitis.	Intervention: oral hygiene instruction and SRP (with or without flap surgery). Control: No periodontal treatment	HbA1C, CRP and adverse events related to periodontal treatment.	9 RCT	Risk of bias in the studies included was high, mostly in the blind.	HbA1c (%): WMD: −0.56 [−0.75; −0.36] *I*^2^: 0%	Publication bias: only articles in English included. Search strategy only included MeSH terms (which may exclude studies indexed in the last 6 months). Only two of the three minimal databases were searched and no gray literature search was performed.

*RCT, Randomized clinical trial; HbA1c, Glycated hemoglobin; FPG, Fasting plasma glucose; OGTT, Oral glucose tolerance test; DM1, diabetes mellitus type 1; DM2, diabetes mellitus type 2; WMD, Weighted mean difference; CP, Chronic periodontitis; SRP, Scaling and root planning; CCT, Controlled clinical trial design; NT, No treatment; CRP, C-reactive protein*.

Finally, in the question: “In diabetic patients, does the exposure to a higher level of HbA1c, compared to stable levels, increase the risk of infectious complications?,” the search strategy applied in EPISTEMONIKOS obtained 7 systematic reviews, and only one of them was relevant for the structured question asked. On the other hand, the search strategy applied in MEDLINE for the same question obtained 9 systematic reviews, and only one of them answered the question, being the same identified with the strategy in EPISTEMONIKOS. The only systematic review answering the question is a qualitative systematic review where no meta-analysis of the studies included was carried out (Tables 3, 5). Besides, this systematic review has methodological limitations, since no exhaustive search in more than one database was carried out and it presented publication bias, since only publications in English were included [36].

**Table 5:** Description of reviews included in question 4.

**Review**	**Inclusion criteria**	**Population**	**Intervention/control**	**Outcomes**	**Studies included**	**Risk of bias, studies included**	**Results**	**Observations**
Rollins et al. [36]	Studies were selected if they included patients with diabetes and HbA1c levels measured within 3 months prior to surgery and if the study reported at least one postoperative outcome. Studies were excluded if they analyzed results based on HbA1c without distinguishing between patients with and without diabetes, if they duplicate data from another included study, or if they did not include any relevant clinical outcome measure (postoperative morbidity and mortality, length of hospital stay, readmission rates and reoperation). Studies reporting the results of a population where not all patients were treated surgically were also excluded.	Adult patients with diabetes				GRADE methodology is used inappropriately to determine risk of bias. Applies criteria to determine certainty of evidence to primary studies (certainty of evidence applies to evidence synthesis).	Qualitative systematic review (without meta-analysis), so they only describe the results of the included articles. Preoperative glycemic control did not influence mortality at 30 days. There were no significant differences in the incidence of stroke, venous thromboembolic disease, hospital readmission and stay in the ITU based on glycemic control. Most studies suggested that there is no relationship between preoperative HbA1c levels and acute kidney injury or the need for postoperative dialysis, dysrhythmia, infection not related to the surgical site, and total length of hospital stay. The literature was highly variable with respect to myocardial events, surgical site infection, and reoperation rates.	Publication bias: articles only in English, does not comply with search in minimum number of databases.

### Methodological Quality of the Reviews

Regarding the methodological quality of the different reviews, the vast majority presented publication bias, because their search was limited to studies published in English. Furthermore, the heterogeneity reported by the different meta-analyses was relatively high, reaching 84% [34]. This accounts for possible differences in the population considered in the different primary studies, since the intervention used throughout the studies was always the same.

The internal validity of the randomized clinical trials was not assessed in all the systematic reviews. However, those that did use an appropriate tool to assess risk of bias reported a high risk in most of the articles included (especially for blinding, selective reporting, and incomplete data extraction).

## Discussion

The aim of the present study was to identify and describe the best available evidence for the treatment of periodontitis in patients with decompensated diabetes and thereby develop a treatment protocol.

The search strategy involved the databases MEDLINE and EPISTEMONIKOS in which the latter focuses on decision-making algorithms in the health area [37]. A remarkable feature of EPISTEMONIKOS is the amount of relevant evidence displayed due to the integration of different other databases. The major benefit is the reliable evidence available to answer health questions based on systematic reviews [37]. In the present study four questions were raised. Theoretically, once these questions had been addressed and answered, it would allow us to propose a treatment protocol for patients with decompensated diabetes and periodontitis that could be implemented in primary health care. However, the present study revealed a lack of evidence precluding the construction of a specific protocol.

In decompensated diabetic patients exposed to periodontal treatment, the search strategy failed to identify any appropriate observational study or systematic reviews regarding the risk of infectious or systemic complications. It can therefore, be concluded that there is lack of scientific information to answer the question. More studies are warranted to determine the proposed relationship whether there is a risk of infectious or systemic complication following periodontal therapy.

As for the question of whether antibiotic prophylaxis reduces infectious complications in decompensated diabetic patients, none of the articles directly addressed the question. The search strategy showed an extensive evidence oriented to the use of antibiotics to improve periodontal parameters instead [38]. Therefore, more randomized clinical trials are needed to assess whether the antibiotic treatment or prophylaxis reduces the infectious complications in decompensated diabetic patients.

With respect to the possible HbA1c decrease in diabetic patients following periodontal therapy, the available evidence revealed a significant reduction of HbA1c after periodontal treatment. This is of clinical relevance since a decrease of 1% in HbA1c reduces the probability of major complications of diabetes by 35% [39]. This reduction has been confirmed in a recent study [40]. A previous systematic review on the other hand indicated a reduction of only 0.29% in HbA1c [17]. These differences could be attributed to the great heterogeneity and the high risk of bias of included articles [41]. It is worth noting that it is still unclear how long the HbA1c reduction lasts after periodontal treatment [17, 40].

Other considerations that may have influenced the results were the inclusion of articles only written in English as well as the non-use of all available databases. Furthermore, there were inconsistencies in the results between most reviews. This might be explained by the moderate to high level of heterogeneity (> 40%) found as well as the broad inclusion criteria, the different follow-ups and other confounding variables that were not considered [33]. While Simpson et al. [17] found a HbA1c reduction of about 0.3%, Baeza et al. [35] found a reduction up to 0.6% following periodontal therapy. In a recent clinical study [42], the effects of periodontal therapy in 100 patients with type 2 diabetes and chronic periodontitis were evaluated. The participants were classified as having good (*n* = 48) or poor (*n* = 52) glycemic control. The study revealed that periodontal treatment decreased the HbA1c levels by 10% in all patients. However, the improvements were more pronounced in patients with worse glycemic control [42].

With respect to the question whether higher levels of HbA1c increase the rate infectious complications, only one systematic review was found [36]. The authors concluded that there was no relationship between the levels of HbA1c and any postsurgical outcome, including infectious complications. It should be noted, however, that the aforementioned review was merely qualitative and a quantitative data synthesis was not performed. Moreover, the included studies only considered patients in secondary care and limited medical specialties (orthopedics and cardiology) thereby omitting primary health care and other specialties which may have influenced the reported results [36].

In general, most of the reviews included studies with high risk of bias which should be considered when interpreting the reported data. The disagreements found between the systematic reviews, on the other hand, might be attributed to the heterogeneity of the studies, because of the presence of different types of adjuvants, follow-up periods and a high presence of biases. It is important to mention the multifactorial nature of the reduction of glycemia, where, for example, oral care instructions alone have been shown to have effects on glycemia [43]. Jain et al. [34] review stands out [34], where only studies of periodontal treatment as monotherapy were included, reducing the heterogeneity of the results with a decrease of 0.26% in HbA1c, similarly to what Simpson et al. [17] exposed [17].

One strengths of the present study is the use of EPISTEMONIKOS and MEDLINE databases. Nevertheless, and despite the thorough and comprehensive search only limited evidence was found. This clearly indicates that more clinical studies are needed. The present study has some of limitations which should acknowledge. The search strategy involved only 2 databases. Although EPISTEMONIKOS is a broad evidence-based healthcare database, the fact that some studies found in MEDLINE were not included in EPISTEMONIKOS [31, 32] shows the probability to exclude some studies for not researching in more databases. Furthermore, the restricted number of primary studies included limits the generalization of the present findings.

Overall, and due to the lack of clinical studies, clinical recommendations cannot be made. More studies are needed to determine the effect of glycated hemoglobin o the risk of infectious complications. A defined HbA1c threshold for dental and periodontal treatment in diabetic patients has yet to be determined. More studies are required, for the establishment of an evidence-based protocol for patient with decompensated diabetes and periodontitis.

## Conclusion

In decompensated diabetic patients, there is lack of scientific information about risk of infectious or systemic complications as a result of periodontal treatment and about the impact of antibiotic treatment or prophylaxis on reduction if infectious complications. A defined HbA1c threshold for dental and periodontal treatment in diabetic patients has yet to be determined. Finally, periodontal treatment does have an impact on HbA1c levels.

## Data Availability Statement

The raw data supporting the conclusions of this article will be made available by the authors, without undue reservation.

## Author Contributions

MD, AM, NH, MR, YY, CS, GJ, JC, JV, and JG: conception and design of the study, literature search, analysis and interpretation of data collected, drafting of article and/or critical revision, final approval, and guarantor of manuscript. MD, FC, MB, FS, and FV-V collected data. JV monitored the process. All authors contributed to the article and approved the submitted version.

## Conflict of Interest

The authors declare that the research was conducted in the absence of any commercial or financial relationships that could be construed as a potential conflict of interest.
